# Dual-band dual-polarized sub-6 GHz phased array antenna with suppressed higher order modes

**DOI:** 10.1038/s41598-024-56218-8

**Published:** 2024-03-13

**Authors:** Debaprasad Barad, Jogesh Chandra Dash, Debdeep Sarkar, P. Srinivasulu

**Affiliations:** 1 Electrical Communication Engineering, Indian Institute of Science(IISc), Bangalore, Karnataka 560021 India; 2 Department of Electronics and Communication Engineering, NIT, Rourkela, Odisha 769008 India; 3 RF and Radar Group, Astra Microwave Private Limited, Hyderabad, Telangana 501218 India

**Keywords:** Engineering, Electrical and electronic engineering

## Abstract

This manuscript proposes a dual-band dual-polarized 64-element phased array antenna (PAA) with suppressed higher order modes (HoMs). Each array element is a microstrip patch structure with complementary split ring resonator (CSRR) loading, allowing for simultaneous dual-band and dual-polarization performance at 3.45 GHz and 3.87 GHz. The proposed PAA exhibits impedance matching of $\le - 20$ dB at 3.45 GHz and 3.87 GHz while limiting the mutual coupling below $- 19$ dB between the adjacent elements at both the operating bands. The proposed design includes a plated through hole placed near the CSRR loading to suppress any higher-order modes (HOMs). Two antenna prototypes, without and with HOMs suppression techniques are fabricated and measured. In addition, we demonstrate the beam steering of the proposed PAA up to $\pm 60^0$ using an off-the-shelf 6-bit phase shifter module. The proposed 64 -element array achieves a gain of 23 dBi, and shows a minimal steering loss of $\simeq 3$ dB over the steering angle.

## Introduction

The future wireless communication technology is expected to use intelligent metasurfaces, massive beamforming for improved user experience, potentially providing dedicated links to individual users^1–5^. This communication technology can be fulfilled using a phased array antenna (PAA), which provides multiple beams with high-speed and precise beam forming, beam steering in azimuth, and elevation plane over a wide angular coverage^6,7^. The mmWave beam forming technique using PAA is discussed in^8–10^. However, because of strict line-of-sight (LOS), prone to signal degradation due to natural obstructions like trees, buildings, and poor signal penetration capabilities in the mm-wave frequency band (FR2), researchers in academia and industry are focused on the realization of base station antenna in FR1 frequency band^11,12^. Several works discuss the 5G base station antenna using the phased array technique,^13,14^, operating at single resonance and single polarized. However, the upcoming frequency standards in future communication technology, such as 5G require multi-band PAAs, which can save cost and space for additional base stations^15^. On the other hand, the multi-path fading in the base station communication system is a critical parameter to study, which can be addressed using the pattern diversity method with a dual-polarization^16^.

A dual-band dual-polarized antenna using modified mushroom unit cell^17^, double loop dipole antenna with metasurface^18^, differential-fed with a microstrip line with open-circuit stepped-impedance resonators^19^, a massive metamaterial loaded MIMO antenna^20^ for sub-6 GHz base station applications are discussed. In sub-6 GHz, a $4\times 4$ array configuration using wideband spiral antenna demonstrates beam steering of $\pm 40^0$^21^. Few designs report the baffles shape with FSS and ferrite loading^22^, bent dipole embedded with two individual microstrip balun^23^, produces dual-band, dual-polarized 5G base station arrays. A butler matrix embedded antenna array^24^, demonstrates fixed beam steering up to $\pm 40^0$. In^25^, a dual-band dual-polarized multi-beam antenna configuration is demonstrated for $\pm 40^0$ fixed beam steering. A dual-polarized $10\times 10$ PAA using a cavity-backed patch embedded with vertical orthogonal balun shows beam steering up to $\pm 45^0$ is discussed in^26^. However, the cavity-like structure of microstrip antenna is very common for higher order modes (HOMs) excitation. The HOMs accelerate the EMI/EMC issues and give rise to spurious harmonic generation in the RF and communication system of the base station unit leading to radiation pattern distortion, and undesirable side lobe level (SLL). In addition to these unwanted problems, due to energy distribution in the unwanted HOMs, the main beam gain of the array decreases when the main beam of the phased array scans to the larger angles. Moreover, when the unwanted SLL is more, the array can not scan the large angle^27^. To the best of the author’s knowledge, a dual-band dual-polarized massive PAA with wide-angle beam steering and suppressed HOMs for sub-6 GHz is not available in open literature.

This article proposes a massive ($8\times 8$) dual-band, dual-polarized PAA using CSRR loading for sub-6 GHz 5G base station applications, where a plated through hole (PTH) is used to suppress the HOMs while improving the impedance matching. Further, the beam steering performance is demonstrated up to $\pm 60^0$ using Ansys HFSS Electromagnetic solver as well as in measurement using a $6-$bit phase shifter module.

## Methods

### Design of dual-band dual-polarized $8\times 8$ PAA with reduced HOM

**Figure 1:**
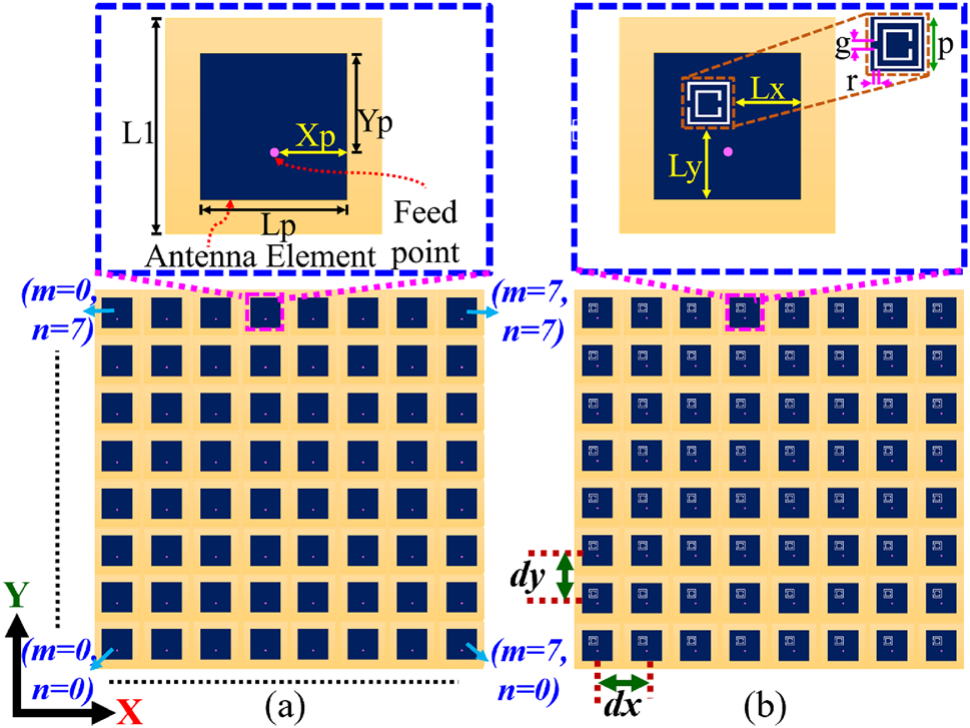
Proposed $8\times 8$ PAA configuration with a zoomed view of the microstrip patch element: (**a**) without CSRR loading, (**b**) with CSRR loading. Dimensions: $L_1= 24$ mm, $L_p= 20$ mm, $X_p= 10$ mm, $Y_p= 6.55$ mm, $L_x= 9.5$ mm, $L_y= 10$ mm, $g=r= 0.4$ mm, $p= 6$ mm, $dx=dy=d=39$ mm, *m*, *n* are the element position.

**Figure 2:**
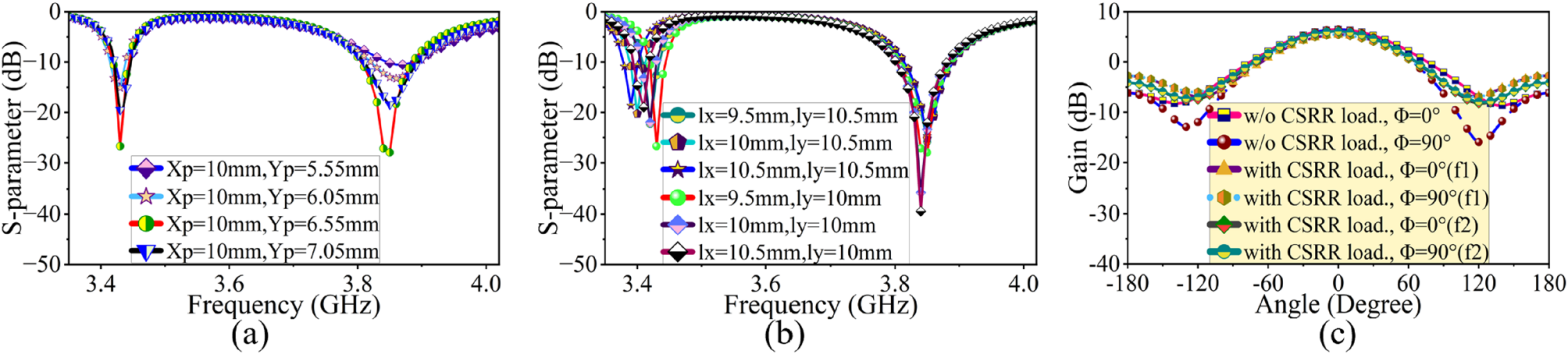
Sensitivity analysis of the CSRR Loaded patch antenna (**a**) by changing the excitation position (**b**) by changing the CSRR location (**c**) single element radiation pattern w/o and with CSRR loading.

**Figure 3:**
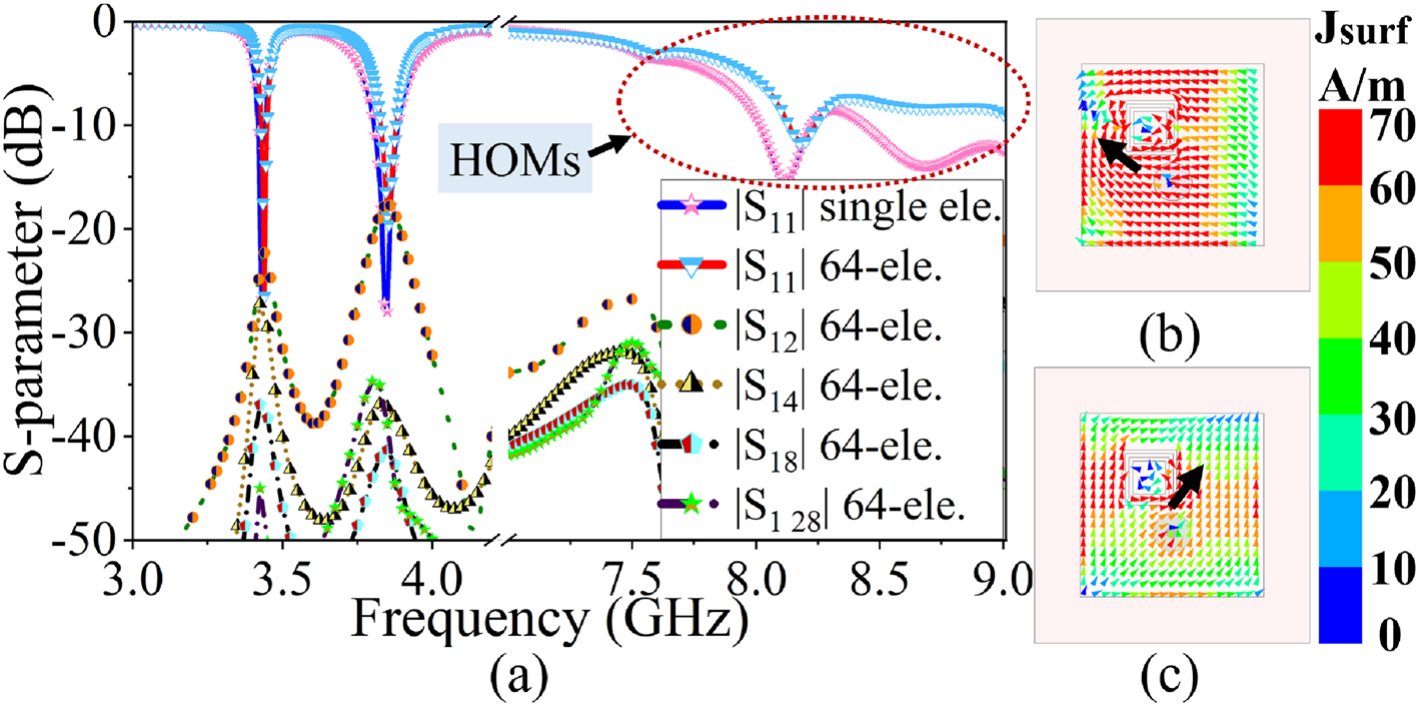
(**a**) S-parameter of the $8 \times 8$ PAA without PTH, current distribution of $m=n=0$ element at (**b**) 3.45 GHz, (**c**) 3.87 GHz.

The proposed dual-band dual-polarized $8 \times 8$ antenna is designed on a single-layer dielectric substrate (Rogers 3003), with $\epsilon _r=3$, tan$\delta =0.001$ and 1.52 mm thickness. Here, an individual antenna element is a square microstrip patch antenna ($L_{p} \times L_{p}$) with CSRR loading (see Fig. 1). The CSRR has a dimension of $p\times p$ with a split gap (g) and ring width of (r) and is etched out from the top surface of the antenna radiator as shown in Fig. 1b. The CSRR at the top surface is a high Q-factor resonator, which exhibits capacitive and magnetic coupling through the ring slot and split gap respectively. This coupled field between the CSRR and patch exhibits dual-band response without affecting primary radiation pattern^28,29^. Next, the excitation and CSRR positions are optimized thoroughly in the HFSS full-wave solver (see Fig. 2). The S-parameter plot, in Fig. 3a, shows the dual-band response at 3.45 GHz and 3.87 GHz of the proposed design. In order to achieve proper functionality of PAA with low mutual coupling, the antennas are spaced at a distance of $0.5\lambda _0$, where $\lambda _0$ is the free-space wavelength at 3.87 GHz operating frequency. This spacing allows for mutual coupling between adjacent elements to be less than or equal to $-19$ dB, with impedance matching of less than or equal to $-20$ dB at both resonances (see Fig. 3a). The polarization state of the primary CSRR-loaded square patch element (i.e. $m=n=0$) in $8\times 8$ PAA is described by the simulated surface current distribution at two operating frequencies (see Fig. 3b,c). The proposed antenna has mutually orthogonal field directions at $\phi =135^0$ and $\phi =45^0$ for 3.45 GHz and 3.87 GHz, representing a dual polarization state (refer to Fig. 3b,c). In addition to the dual-band and dual-polarization state, the proposed antenna loaded with CSRRs also exhibits undesired higher-order modes, as shown in the S-parameter response in Fig. 3a.

### Configuration of dual-band massive antenna array with HOMs suppression technique

**Figure 4:**
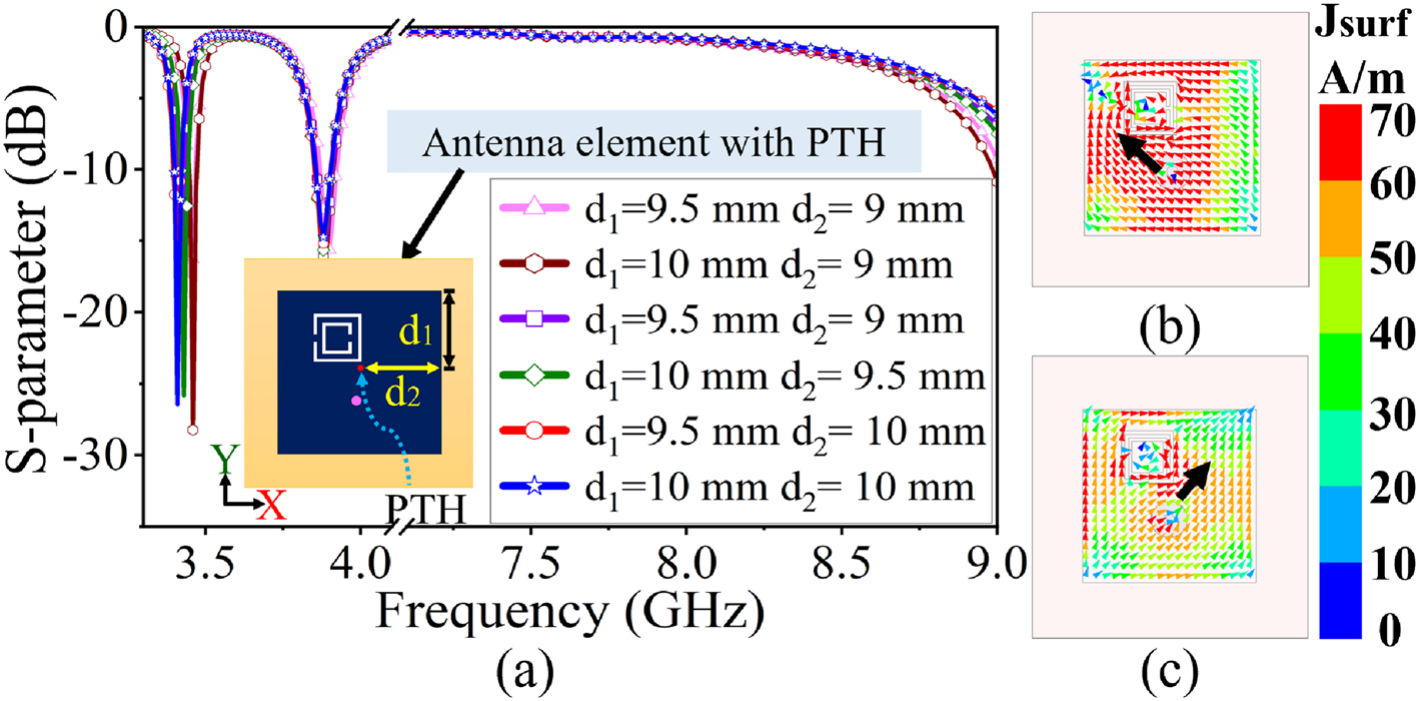
(**a**) Optimization of S-parameter by changing PTH position, current distribution at (**b**) 3.45 GHz, (**c**) 3.87 GHz.

**Figure 5:**
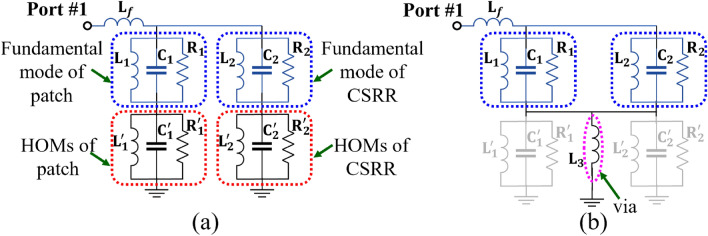
Equivalent circuit model of (**a**) patch with CSRR loading, (**b**) patch with CSRR loading and PTH.

In the proposed microstrip antenna loaded with CSRR, the unwanted higher-order mode (HoM) is suppressed by placing a PTH near the CSRR. The location of the PTH is determined through a parametric study, as demonstrated in the S-parameter plot shown in Fig. 4a. Figures 4b,c display the simulated surface current distribution along $\phi =135^0$ and $\phi =45^0$ at 3.45 GHz and 3.87 GHz, respectively, for the CSRR-loaded antenna in the presence of PTH. The presence of PTH does not interrupt operating frequencies or surface current distribution. The PTH suppresses the HOMs present in the X-band region ($4-9$ GHz) while improving the dual-band impedance matching (see Fig. 4a). In order to provide a clearer insight into HOMs suppression, a simplified approximate circuit model^28–30^ of the patch with CSRR loading and CSRR loaded patch with via is presented in Fig. 5a,b respectively. Here, the co-axial excitation is represented as the inductive probe ($L_f$). The patch is represented as the RLC resonator ($R_1, L_1, C_1$) and the CSRR loading is represented as a shunt RLC resonator ($R_2, L_2, C_2$) which exhibits a low resonance frequency compared to conventional patch shown in Fig. 1a. Similarly, the HOMs of the patch and CSRR are represented as separate RLC resonators ($R^{'}_{1,2}, L^{'}_{1,2}, C^{'}_{1,2}$), as shown in Fig. 5a, responsible for the unwanted auxiliary radiation. Figure 5b presents a CSRR-loaded patch with a via ($L_3$), which routes the fundamental radiating mode current through a short circuit path to ground thereby suppressing the HOMs.

**Figure 6:**
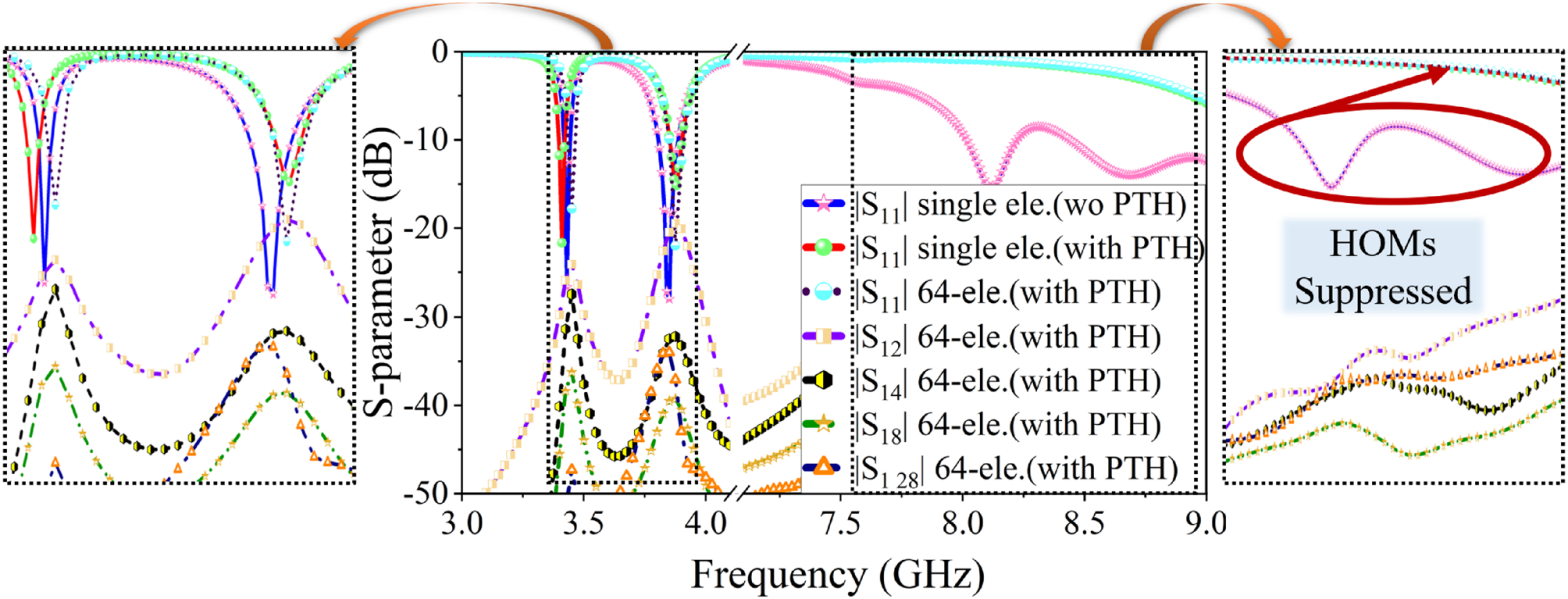
S-parameter of the $8 \times 8$ PAA with suppressed HOMs.

Next, the element with suppressed HOMs is used to configure $8 \times 8$ antenna array with an inter-element spacing of $0.5\lambda _0$. The S-parameter response of the $8 \times 8$ antenna array is studied in Fig. 6. It is observed that the mutual coupling between antenna elements in the array is below $-20$ dB, regardless of the antenna position in the array (see Fig. 6).

### PAA beam steering performance

**Figure 7:**
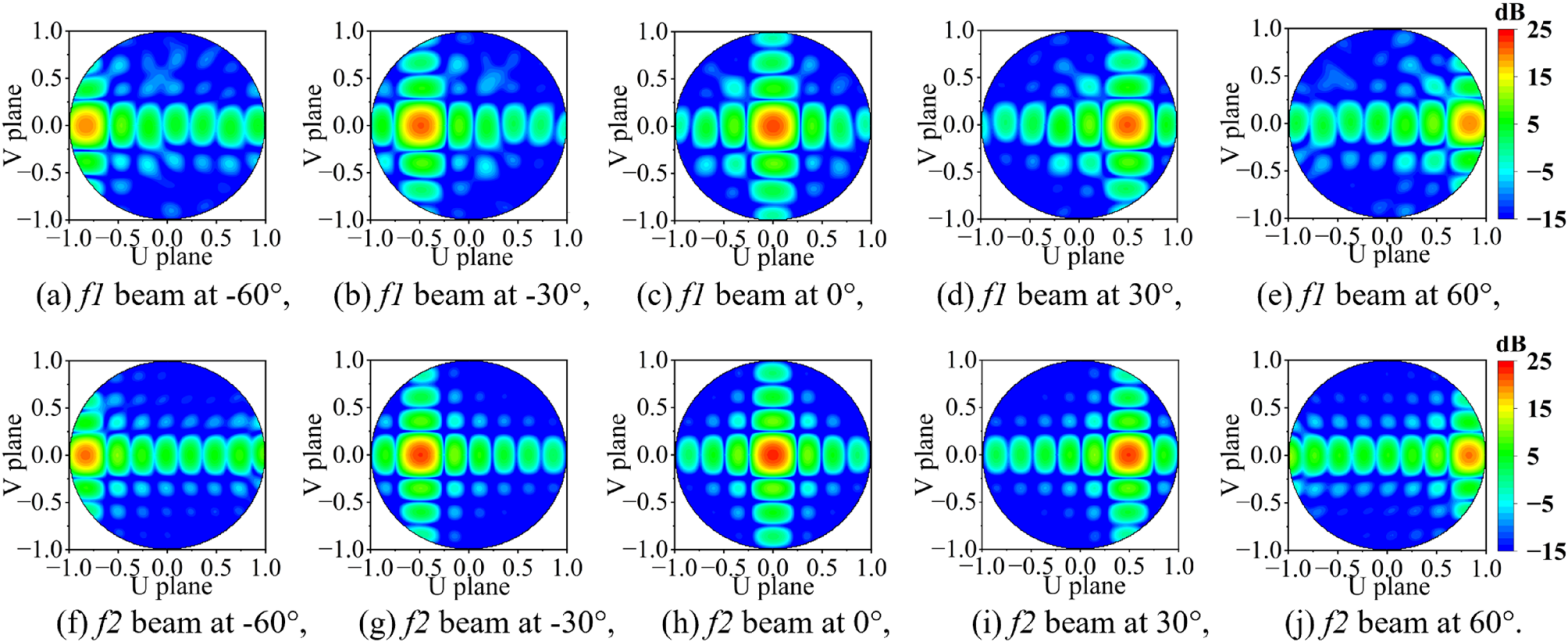
Visualization of 3D beam steering radiation pattern of 64-Element array in contour plot (**a**–**j**) $-60^0$ to $60^0$, at $f_1$=3.45 GHz, $f_2$=3.87 GHz.

The 5G base station technology demonstrates three sector deployment structure with 3D sectorization of the beam pattern in azimuth and elevation, where each sector is equipped with a beam-forming antenna module (usually a PAA) to provide an angular range of $120^0$ (i.e $\pm 60^0$)^31,32^. In this scenario, the PAA steers the beam by controlling the aperture distribution. This is achieved by compensating the progressive phase shift in the array factor (AF) of the planar $M\times N$ array and represented in Eq. (1)^33^.

1 $$\begin{aligned} \begin{aligned} AF = I_0 \Sigma ^{M}_{m=1} e^{j(m-1)(k_0d_x \sin {\theta }\cos {\phi }+\beta _x)} \times \Sigma ^{N}_{n=1} e^{j(n-1)(k_0d_y \sin {\theta }\sin {\phi }+\beta _y)} \end{aligned} \end{aligned}$$

where, $I_0$ is the uniform excitation amplitude for all *m* and *n* elements, $k_0$ is the wave number. The common main beam of PAA is in the direction of $\theta =\theta _0$ is the beam steering angle and $\phi =\phi _0$ is the plane of the beam steering. The principal maximum of the main beam is specified by $(\theta _0, \phi _0)$. To steer the main beam from the broadside, the element steering phase ($\beta _x$, $\beta _y$) is summed at each element (*m*, *n*) for providing a combined phasing ($\Delta ph$) at the $mn^{th}$ element using Eqs. (2)–(4). Moreover, the steered beam is controlled by active element pattern^33,34^.

2 $$\begin{aligned} \beta _x= & {} -k_0d_x \sin {\theta _0}\cos {\phi _0} \end{aligned}$$

3 $$\begin{aligned} \beta _y= & {} -k_0d_y \sin {\theta _0}\sin {\phi _0} \end{aligned}$$

4 $$\begin{aligned} \Delta ph= & {} (m \times \beta _x) + (n \times \beta _y) \end{aligned}$$

**Figure 8:**
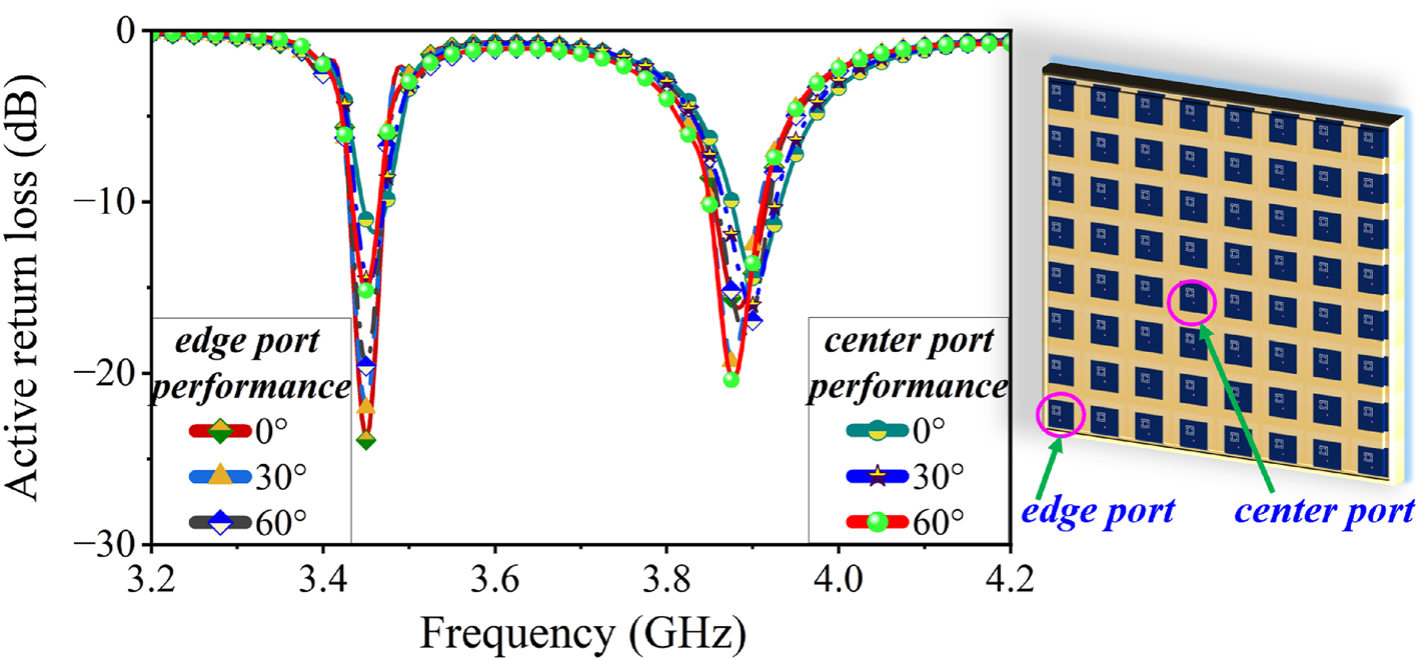
Active return loss of the $8\times 8$ PAA during beam steering analysis.

The active element pattern can be reshaped by controlling the mutual coupling between the elements. This is possible with increasing the inter-element spacing while maintaining the maximum peak gain and good side lobe levels, but large spacing raises the grating lobe issues. The element spacing is a trade-off between the grating lobe and mutual coupling issues in PAA^35^. For beam steering upto $\pm 60^0$, the PAA must satisfy the Eq. (5) at the highest operating frequency to confirm a single main lobe and no grating lobe in $-\pi /2<\theta <\pi /2$ region. Further, the PAA with N elements restricts the Eq. (5) by $(N-1)/N$ and the 3 dB beam width of the antenna array^34^.

5 $$\begin{aligned} \frac{d}{\lambda _0} \le \left( \frac{1}{1+\sin \left( \theta _0 \right) } \right) \end{aligned}$$

Here, the proposed $8\times 8$ PAA uses $0.5\lambda _0$ spacing to minimize the mutual coupling up to $\le -19$ dB and achieves a beam steering up to $\pm 60^0$. The beam steering performance is demonstrated using HFSS EM solver by exciting each element with a uniform amplitude and phase coefficient (using Eq. 4)^36–38^. The solver calculates the absolute phase coefficient for each element and steers the main beam to the desired angle. The 3D beam steering pattern is presented using contour plots at $30^0$ step (see Fig. 7). A minimum steering loss close to 3 dB is achieved by optimizing the active element for a stable radiation pattern on the order of $\cos ^{1.25}{\theta _0}$^39^. In addition to this, the proposed $8\times 8$ PAA shows a consistent active return loss of $\le -12$ dB (see Fig. 8), which ensures no scanning blindness during the beam steering performance up to $\pm 60^0$.

## Experimental characterization of dual-band dual-polarized $8\times 8$ PAA with reduced HOM

**Figure 9:**
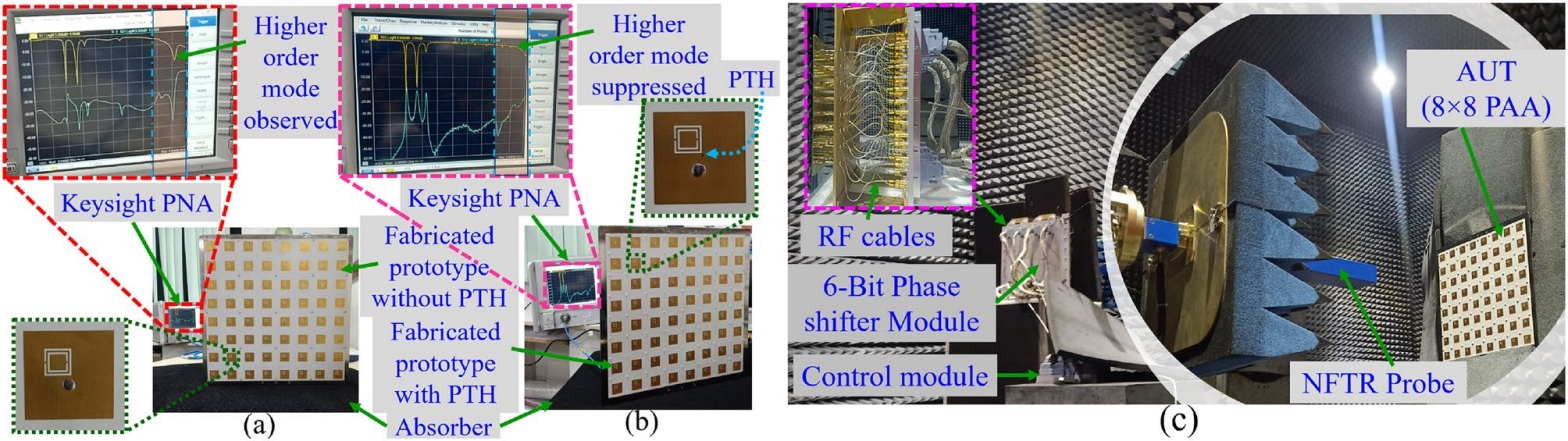
Measurement setup for S-parameter characterization, (**a**) without PTH, (**b**) with PTH, (**c**) beam steering characterization of the fabricated $8\times 8$ PAA.

**Figure 10:**
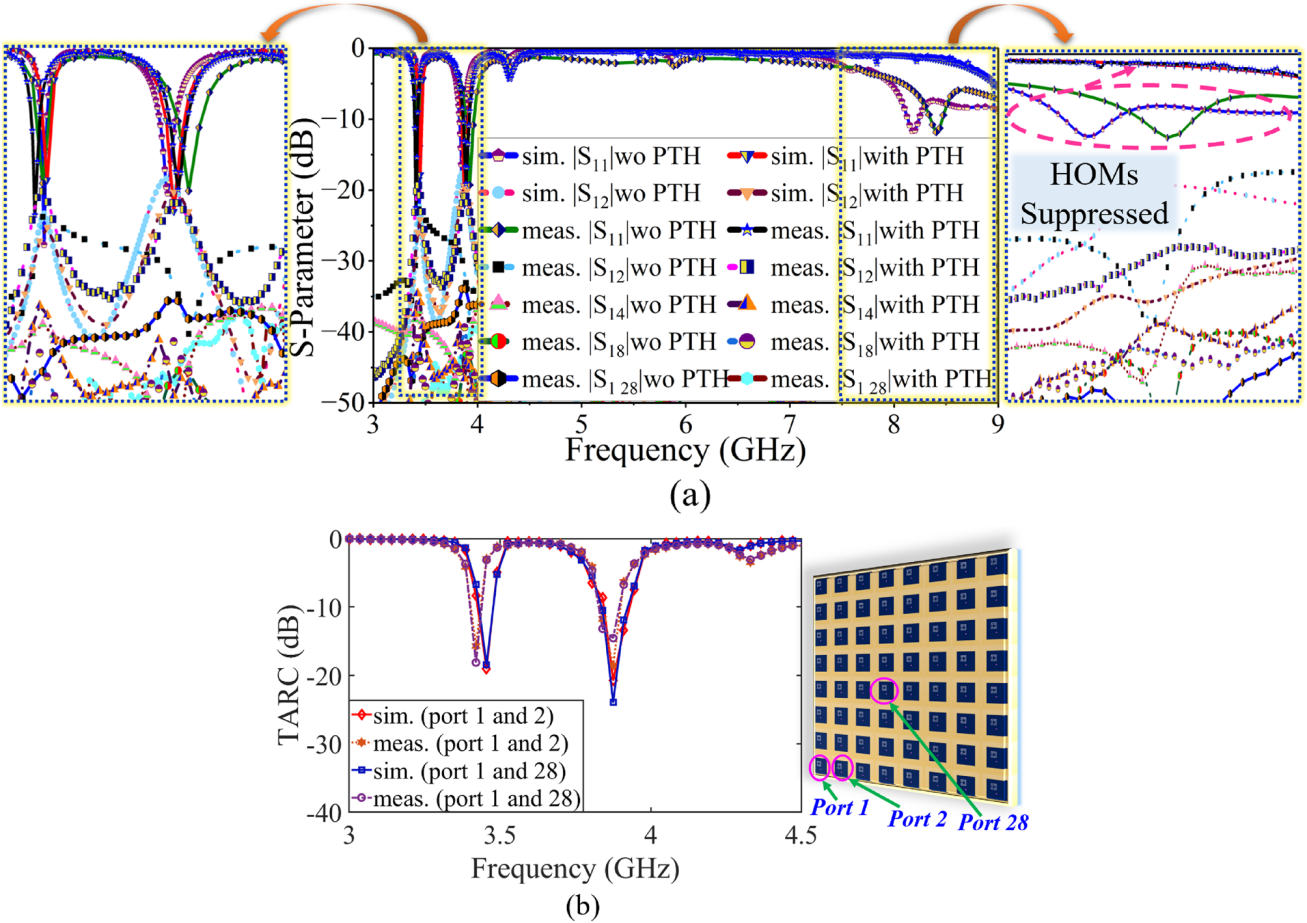
(**a**) Comparison of S-parameter result of $8\times 8$ PAA without and with HOMs suppression, (**b**) comparison of TARC result.

The proposed massive array without and with HOMs suppression is fabricated. The fabricated PCB and the co-axial connector (SMA-F) are assembled on a metal plate. First, the fabricated prototype is validated through S-parameter measurement (see the test setup in Fig. 9a,b). Here, the measurement is conducted using Keysight PNA. The port-1 and port-2 of the PNA are connected to port-1 and port-2 of the AUT while terminating the remaining ports with $50 \Omega$ load. The measured S-parameter results show both the prototypes achieve dual-band resonance with impedance matching of $\le -20$ dB and mutual coupling of $\le -19$ dB and are in good agreement with the simulated S-parameter result (see Fig. 10a). In addition to this, the total active reflection coefficient (TARC) of $\le -15$ dB is achieved at both the operating frequency (see Fig. 10b), which assure efficient operation in active mode^28^.

**Figure 11:**
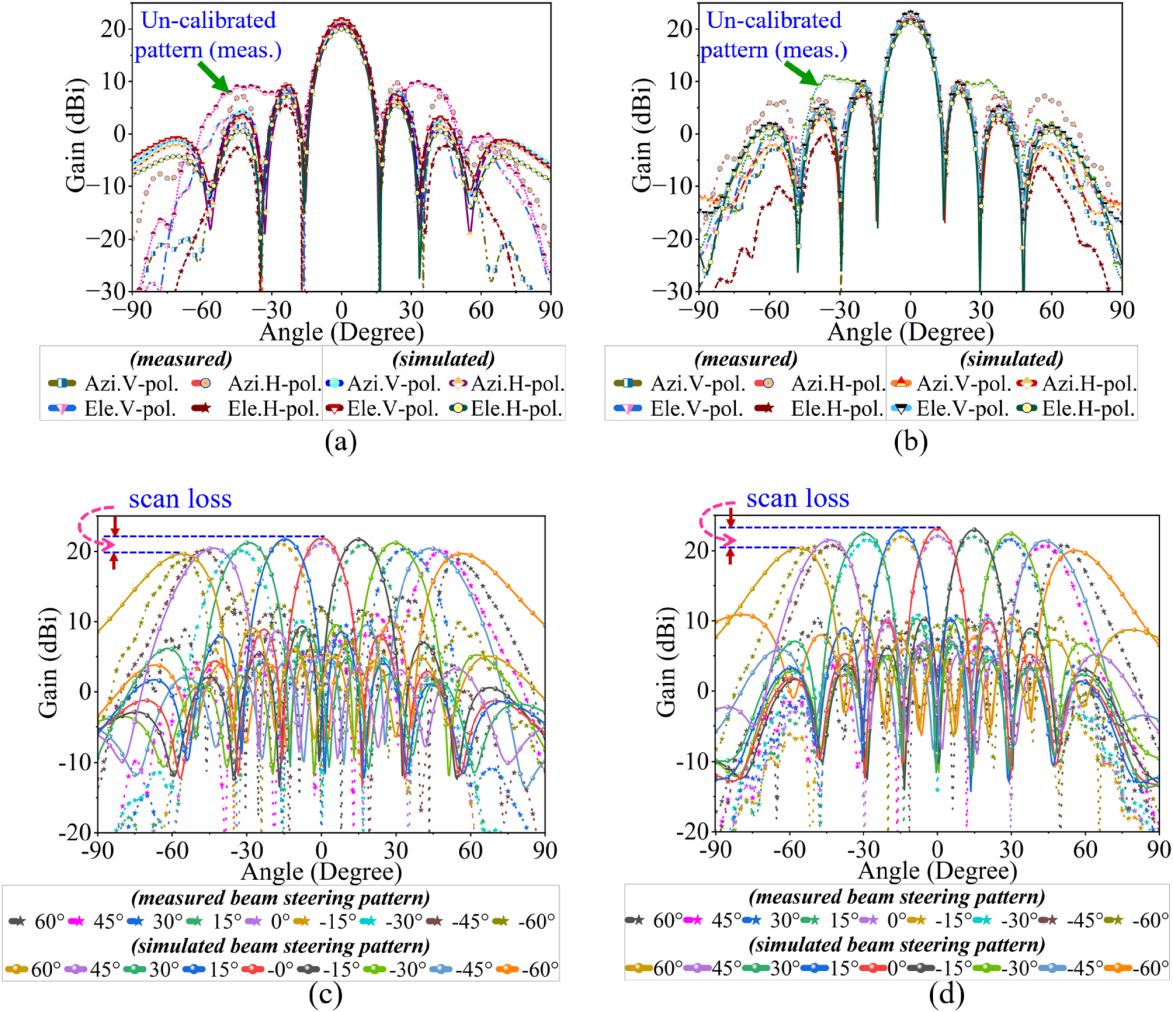
Comparison of the measured radiation pattern with simulation, boresight (**a**) 3.45 GHz, (**b**) 3.87 GHz, beam steering (**c**) 3.45 GHz, (**d**) 3.87 GHz.

Next, the radiation pattern of the proposed CSRR-loaded massive antenna array is measured in the near field test range (NFTR). The proposed antenna array is integrated with an RF phase shifter and control module with RF cables (see Fig. 9c). The phase shifter module incorporates a 1 : 32 way power divider (PD) network, where each output port of PD has a 6-bit digital phase shifter and RF switch to enable phase control of each element, which is commercial off-the-shelf hardware of M/s Astra microwave product ltd (2 phase shifter modules are used here). The integrated RF module introduces phase errors, which are captured in discrete measurements. The phase errors are normalized using the phase collimation process, where a suitable phase state is applied to each element. First, the boresight un-calibrated pattern is measured. The un-calibrated pattern is not capable of producing a perfect SLL and first null, which is essential to estimate the Rayleigh resolution. Later, the antenna array is calibrated using the phase collimation process. In this, the proposed antenna array plane is transformed to measurement plane in the NFTR acquisition system. The NFTR probe is aligned to the antenna elements as per the measurement plane. The respective phase error of each element is acquired and then corrected by compensating the suitable phase state through the control and phase shifter module^40,41^. This process is repeated for both the resonances to ensure nearly zero beam pointing error and minimum SLL. The calibrated antenna array yields a well-defined SLL ($\simeq 13$ dB) and first null (see Fig. 11a,b). The proposed antenna achieves boresight gain of 21.8 dBi, 23 dBi, and 3 dB beam width ($\theta _b$) of $14^0$, $12.5^0$ at 3.45 GHz, 3.87 GHz respectively and are in good agreement with simulated results. Next, the beam steering patterns are measured at every $15^0$ step (see Fig. 11c,d), by loading the required phase state to the phase shifter module (using equ.4). It is observed, at $\theta _0=60^0$ the 3 dB beam width is increased to $24^0$, $22^0$ at 3.45 GHz, 3.87 GHz respectively (with a factor of $\theta _b/\cos {\theta _0}$). Also, the steered beam located at $\approx 56^0$, $\approx 53^0$ (i.e $\le 60^0$) at 3.45 GHz, 3.87 GHz respectively, which is because of the active element pattern with a factor of $\sqrt{\cos {\theta }}$ and applied progressive phase bits of $\Delta \beta =5.625^0$ corresponds to the 6-bit phase shifter. A comparison study between the available literature and the proposed sub-6 GHz antenna array is presented in Table I. It infers that, a dual-band dual-polarized wide angle beam steering up to $\pm 60^0$ ($\approx 56^0$, $\approx 53^0$ measured) with suppressed HoMs. This shows the superiority of the proposed work compared to published works.

**Table 1:** Comparison of the proposed antenna array with available open literature.

References	Array dimension	**Operating bands**	Beam steering range	HOMs suppression
^21^	$4\times 4$	3.3–5 GHz	$\pm 40^0$	No
^23^	$1\times 4$	2.5–2.69 GHz, 3.3–3.6 GHz	No	No
^24^	$1\times 6$	5.4–5.6 GHz	$\pm 40^0$	No
^25^	$12\times 12$	3.3–4.2 GHz, 5.15–5.93 GHz	$\pm 40^0$	No
^26^	$10\times 10$	3.6–4.4 GHz	$\pm 45^0$	No
This work	${8\times 8}$	3.43–3.48, 3.84–3.92	$\pm {56}^0 \pm {53}^{0}$	Yes

## Conclusion

Here, a 64 element PAA configuration uses a CSRR-loaded antenna element, which facilitates achieving a wide-angle beam steering performance at both resonances. Here, a CSRR loading on a square microstrip antenna is studied for dual-band dual-polarized operation at 3.45 GHz, 3.87 GHz, with improved impedance matching $\le -20$ dB and mutual coupling $\le -19$ dB. The HOMs suppression is achieved by placing a PTH near the vicinity of the CSRR loading. To date, fixed beam steering using Butler matrix circuit-based antennas is demonstrated^24,25^, even a modified cavity technique is reported to enhance the scan performance^26^ by reducing the mutual coupling, and it is limited up to $\pm 45^0$ by the element size. The proposed array configuration uses a CSRR-loaded antenna element, which facilitates achieving a wide angle beam steering performance up to $\approx 56^0$, $\approx 53^0$ at 3.45 GHz, 3.87 GHz respectively. The wide angle beam steering performance is studied using a contour plot and validated by measuring the fabricated prototype. The phase collimation method helps to achieve a well-defined SLL and first null. The proposed antenna array achieves a maximum gain of 23 dBi, while maintaining a minimum scan loss of $\simeq 3$ dB.

## Data Availability

The authors declare that the data supporting the findings of this study are available within the paper.
